# Progressive multifocal leukoencephalopathy during 4 years of Palbociclib for advanced breast cancer with a history of follicular lymphoma patient

**DOI:** 10.1007/s13365-025-01255-w

**Published:** 2025-04-21

**Authors:** Ryosuke Koyamada, Mariko Nishihara, Tetsuhiro Masaki, Tomohito Shimada, Kazuhiro Ishikawa, Jun Hashimoto, Nobuyoshi Mori, Asami Namai, Hideaki Yokoo, Kenta Takahashi, Tadaki Suzuki, Kazuo Nakamichi, Shinichiro Mori

**Affiliations:** 1 Department of Hematology, St. Luke’s International Hospital, Tokyo, Japan; 2 Department of Hematology, Yokosuka Kyosai Hospital, Yokosuka, Japan; 3 Department of Infectious Disease, St. Luke’s International Hospital, Tokyo, Japan; 4 Department of Medical Oncology, St. Luke’s International Hospital, Tokyo, Japan; 5 Department of Pathology, St. Luke’s International Hospital, Tokyo, Japan; 6 Department of Human Pathology, Gunma University Graduate School of Medicine, Maebashi, Japan; 7 Department of Pathology, National Institute of Infectious Diseases, Tokyo, Japan; 8 Department of Virology 1, National Institute of Infectious Diseases, Tokyo, Japan

**Keywords:** JC virus, Progressive multifocal leukoencephalopathy, CDK4/6 inhibitor, Palbociclib, Breast cancer

## Abstract

A 67-year-old woman with a history of follicular lymphoma and recurrent breast cancer with multiple metastasis developed PML during receiving fluvestrant plus palbociclib. Rituximab and bendamustine had finished 6.5 years ago. Discontinuing palbociclib and using mirtazapine, she is alive a year after diagnosis of PML. It is rare for PML to be diagnosed long after the last dose of rituximab and bendamustine. We will discuss the possible involvement of palbociclib in the course of PML.

**Supplementary Information:**

The online version contains supplementary material available at 10.1007/s13365-025-01255-w.

## Introduction

Progressive multifocal leukoencephalopathy (PML) is a progressive and fatal central nervous system (CNS) disease caused by the reactivation of the John Cunningham virus (JCV) in patients with cellular immunodeficiency (Tan and Koralnik 2010). While historically linked to HIV and hematologic malignancies, drug-related PML has been reported in association with natalizumab, dimethyl fumarate, fingolimod, and other agents, including rituximab, alemtuzumab, and mitoxantrone (Berger 2017). To the best of our knowledge, only one other case of PML potentially associated with a cyclin-dependent kinase (CDK) 4/6 inhibitor has been documented in the Food and Drug Administration (FDA)’s digital reporting system (Center for Drug Evaluation and Research 2023). However, the clinical course of PML while using CDK4/6 inhibitors has not been reported in the literature.

## Case presentation

The patient is a 68-year-old woman with a history of treatment for breast cancer and follicular lymphoma. She was diagnosed with breast cancer 17 years ago as pT2N0M0, pStage IIA, and was treated with four cycles of cyclophosphamide, epirubicin, and 5-fluorouracil, followed by postoperative radiation therapy and anastrozole. Four years ago, metastatic recurrence in the lungs and sternum led to the initiation of palbociclib plus fulvestrant. In addition, she was diagnosed with follicular lymphoma 12 years ago as Ann Arbor Stage IIA with a low-risk International Prognostic Index. Her treatment history for lymphoma included four courses of rituximab 11 years ago, eight courses of rituximab combined with dexamethasone pulse 6.5 years ago, and five courses of bendamustine plus rituximab six years ago. She has taken aleviatin 150 mg once daily and levetiracetam 500 mg twice a day for epilepsy.

Five months before admission, she began exhibiting abnormal behavior, including confusion and disorientation. Palbociclib was discontinued at that time. Her symptoms progressively worsened over two months, leading to dizziness, gait disturbances, and slow responses. So, she was admitted to our hospital for further investigation.

**Fig. 1:**
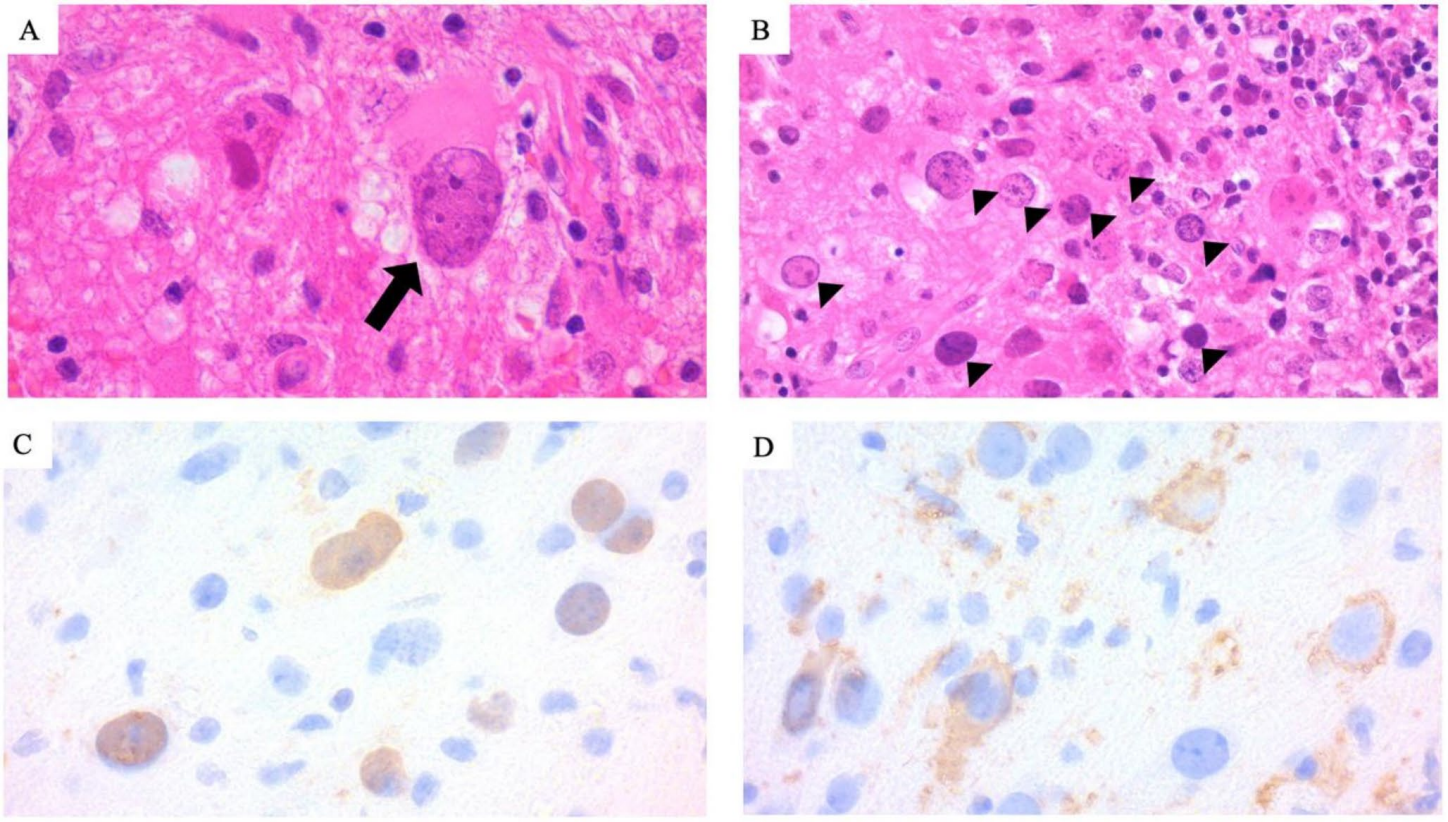
Histopathology of cerebellar biopsy specimen

Brain magnetic resonance imaging (MRI) revealed lesions in the right pons and cerebellum with T1-weighted low and T2-weighted high signals at the time of initial symptoms started. However, at the time of admission, brain MRI showed a high signal area on DWI around the right middle cerebellar peduncle to the pons and medulla oblongata, with high signal on FLAIR with contrast enhancement. Laboratory findings were remarkable for lymphocyte count of 600–800/µL for two years before admission, a CD4:8 ratio of 1.41, IgG was 829 mg/dL. Tumor markers were stable as soluble interleukin-2 receptor levels of 1980 U/mL, CEA was 5.0 ng/mL and CA15-3 was 15.1 U/mL. Anti-HIV antibody, hepatitis B surface antigen, and anti-hepatitis C virus antibody was negative. Cerebrospinal fluid (CSF) analysis showed no elevation of protein level and cell count. Cytology was negative. The cerebellar biopsy revealed demyelination, atypical astrocytes, and enlarged oligodendroglial nuclei (Fig. 1), and the Gunma University Pathology Consultation team confirmed the presence of JCV by polymerase chain reaction (PCR). These findings led to a definitive diagnosis of PML. CSF analysis showed no elevation in protein levels or cell count, and cytology was negative at the initial examination. The cerebellar biopsy revealed demyelination, atypical astrocytes, and enlarged oligodendroglial nuclei (Fig. 1). The Gunma University Pathology Consultation team confirmed the presence of JCV by polymerase chain reaction (PCR), leading to a definitive diagnosis of PML. Although the initial CSF examination showed no findings suggestive of meningitis, the BIOFIRE^®^ FILMARRAY^®^ Meningitis/Encephalitis (ME) Panel (bioMérieux, France) was conducted to confirm the absence of co-infection.

Following the diagnosis, mirtazapine monotherapy was initiated 2.5 months after admission, as mefloquine was contraindicated due to epilepsy.

**Fig. 2:**
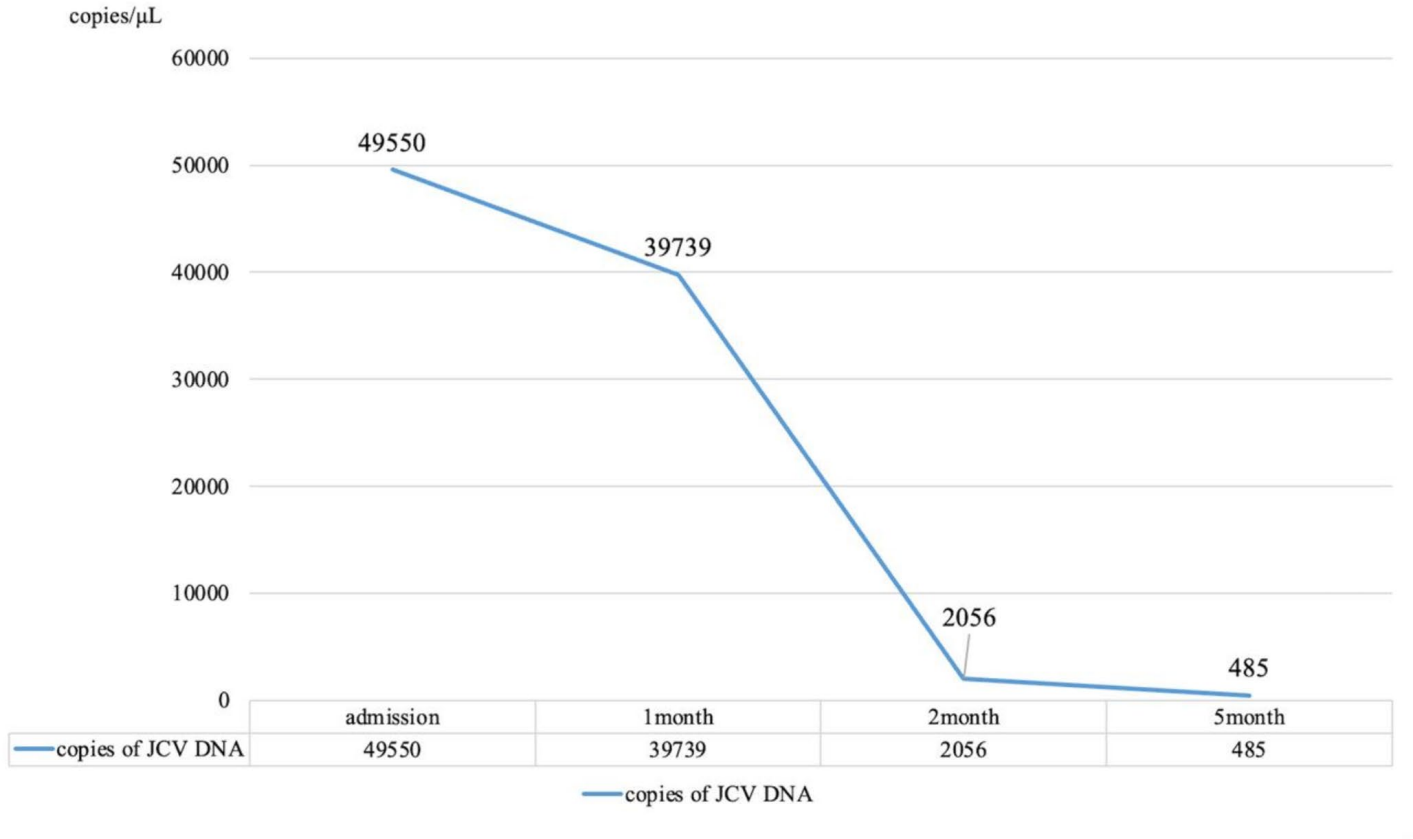
Summary of the results of CSF analysis over three time points

**Fig. 3:**
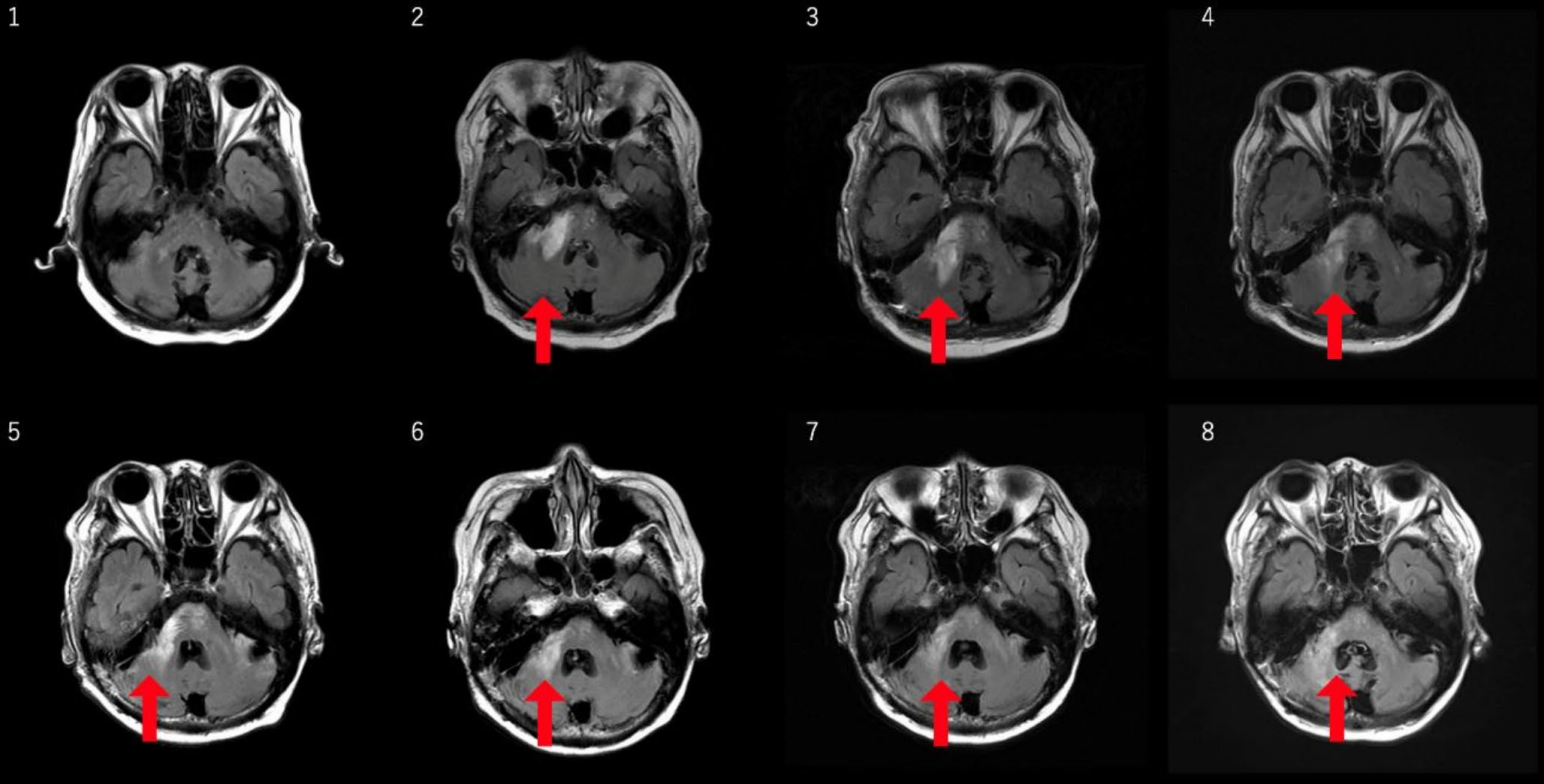
Axial fluid-attenuated inversion recovery (FLAIR) images with contrast enhancement

As for JCV DNA PCR in CSF, it was 49,550 copies/mL at the time of diagnosis. However, it had already decreased to 39,730 copies/mL at the time of starting mirtazapine. It decreased steadily to 2,056 copies/mL after 1 month, and to 485 copies/mL after 3 months. (Fig. 2) Regarding MRI findings, high T2 and FLAIR signals with contrast enhancement were observed around the pons and middle cerebellar peduncle at the time of strating treatment, correlating with the patient’s symptoms. Gradually, the contrast enhancement disappeared, and the FLAIR high areas improved over five months after initiating mirtazapine. (Fig. 3)

Her activities of daily living (ADL) remained impaired. She was discharged to a care facility three months after admission and has been under outpatient follow-up. At one year after diagnosis, MRI and CSF findings continue to show improvement.

## Discussion

The timeline and treatment history of this case provide significant insights. Rituximab and bendamustine are known to cause cellular immunosuppression (García Muñoz et al. 2014). This may have contributed to prolonged lymphopenia and PML onset. However, the interval of six years from the final dose of rituximab and 12 years from the FL diagnosis to the diagnosis of PML is atypical. Previous studies reported that median time of PML onset from final dose of rituximab was 5.5 months (Carson et al. 2009), and with hematological malignancies, median time of PML diagnosis from the diagnosis of hematological malignancies was 48.5 months (Neil and DeAngelis 2017). In addition to that, in the patients of FL treated with bendamustine plus rituximab followed by rituximab maintenance like this case, PML occurred 10 to 31 months from the first dose of BR treatment (D’Alò et al. 2020). In this case, palbociclib, taken for four years before symptom onset, was discontinued, coinciding with a reduction in JCV DNA levels. This suggests a potential role of palbociclib in triggering PML by compounding the immunosuppressive effects of prior treatments.

This case report describes a patient with solid tumors and hematological malignancies who developed PML while receiving a CDK4/6 inhibitor. Recently, cases of posterior reversible encephalopathy syndrome associated with immune checkpoint inhibitors have also been reported (Maur et al. 2012). When abnormal brain MRI findings are observed in patients with malignancies, it is crucial to conduct a comprehensive diagnosis considering the patient’s clinical background and drug history.

Although neutropenia is a known adverse effect in pivotal clinical trial of palbociclib, most infections were non-severe (Turner et al. 2018). Chronic lymphopenia in this patient likely increased susceptibility. A systematic literature review as we conducted, identified two cases of opportunistic infections linked to CDK4/6 inhibitors: pneumocystis pneumonia following palbociclib (Guillaume et al. 2020) and abemaciclib (Ashraf et al. 2022). (Appendix) Additionally, one case of PML with palbociclib was reported on the FDA, lacking detailed clinical information. This is the first case with a detailed clinical course. Future cases should be documented to facilitate data collection and further analysis of the mechanisms triggering PML onset in immunosuppressed patients.

## Conclusion

This case underscores the importance of recognizing potential complications in patients receiving CDK4/6 inhibitors, particularly those with prior exposure to rituximab or bendamustine.

## Electronic supplementary material

Below is the link to the electronic supplementary material.

Supplementary Material 1

## Data Availability

No datasets were generated or analysed during the current study.
