# Correction: Progressive multifocal leukoencephalopathy during 4 years of Palbociclib for advanced breast cancer with a history of follicular lymphoma patient

**DOI:** 10.1007/s13365-025-01259-6

**Published:** 2025-04-28

**Authors:** Mariko Nishihara, Ryosuke Koyamada, Tetsuhiro Masaki, Tomohito Shimada, Kazuhiro Ishikawa, Jun Hashimoto, Nobuyoshi Mori, Asami Namai, Hideaki Yokoo, Kenta Takahashi, Tadaki Suzuki, Kazuo Nakamichi, Shinichiro Mori

**Affiliations:** 1 Department of Hematology, St. Luke’s International Hospital, Tokyo, Japan; 2 Department of Hematology, Yokosuka Kyosai Hospital, Yokosuka, Japan; 3 Department of Infectious Disease, St. Luke’s International Hospital, Tokyo, Japan; 4 Department of Medical Oncology, St. Luke’s International Hospital, Tokyo, Japan; 5 Department of Pathology, St. Luke’s International Hospital, Tokyo, Japan; 6 Department of Human Pathology, Gunma University Graduate School of Medicine, Maebashi, Japan; 7 Department of Pathology, National Institute of Infectious Diseases, Tokyo, Japan; 8 Department of Virology 1, National Institute of Infectious Diseases, Tokyo, Japan

**Journal of NeuroVirology**

10.1007/s13365-025-01255-w

The order of the first two authors is incorrect. Mariko Nishihara should be the first author and Ryosuke Koyamada should be the second author. The order of the other authors is correct.

