# Supplementary material for: Progressive multifocal leukoencephalopathy during 4 years of Palbociclib for advanced breast cancer with a history of follicular lymphoma patient
Source: J Neurovirol. 2025 Apr 21;31(2):191–5. doi: 10.1007/s13365-025-01255-w (PMC12137487; doi:10.1007/s13365-025-01255-w)
Supplement: Supplementary file 1 — Supplementary Material 1 [file 13365_2025_1255_MOESM1_ESM.docx]

**Supplement**

We conducted a systematic literature review to assess the occurrence of opportunistic infections in patients with a history of CDK inhibitor use. The CDK inhibitors included in this review were the four clinically approved drugs: palbociclib, abemaciclib, ribociclib, and trilaciclib.

Opportunistic infections were defined as those occurring in an immunosuppressive state equivalent to the development of PML. The infections considered were based on the definition of clinically significant infections in idiopathic CD4 lymphocytopenia(Lisco et al. 2023). The included infections are as follows: Cryptococcosis, histoplasmosis, disseminated or pulmonary non-tubercular mycobacterial infections, human papillomavirus infections and related diseases, cervical cancer, varicella-zoster virus (VZV) infections, herpes simplex infections, oncogenic viruses, Kaposi’s sarcoma, non-Hodgkin lymphoma, nasopharyngeal carcinoma, gastric carcinoma, molluscum contagiosum, cytomegalovirus (CMV) end-organ disease, CMV infections, Pneumocystis pneumonia, coccidioidomycosis, progressive multifocal encephalopathy.

Three reviewers independently conducted the literature review using the research formula provided in the supplementary file. We searched for literature published after 2000, as clinical trials of the first-generation CDK inhibitors began in 1998. Publication languages other than English and Japanese were excluded from the review. A total of 1,567 articles from PubMed, 5,103 articles from Embase, and 153 articles from Ichushi were identified. (Appendix Figure 1) Only one case met the inclusion criteria and was included in the review (Guillaume et al. 2020). Additionally, an internet-based search revealed one more similar case (Ashraf et al. 2022). The clinical characteristics of the reviewed cases are summarized in Table 1.

Appendix Figure 1


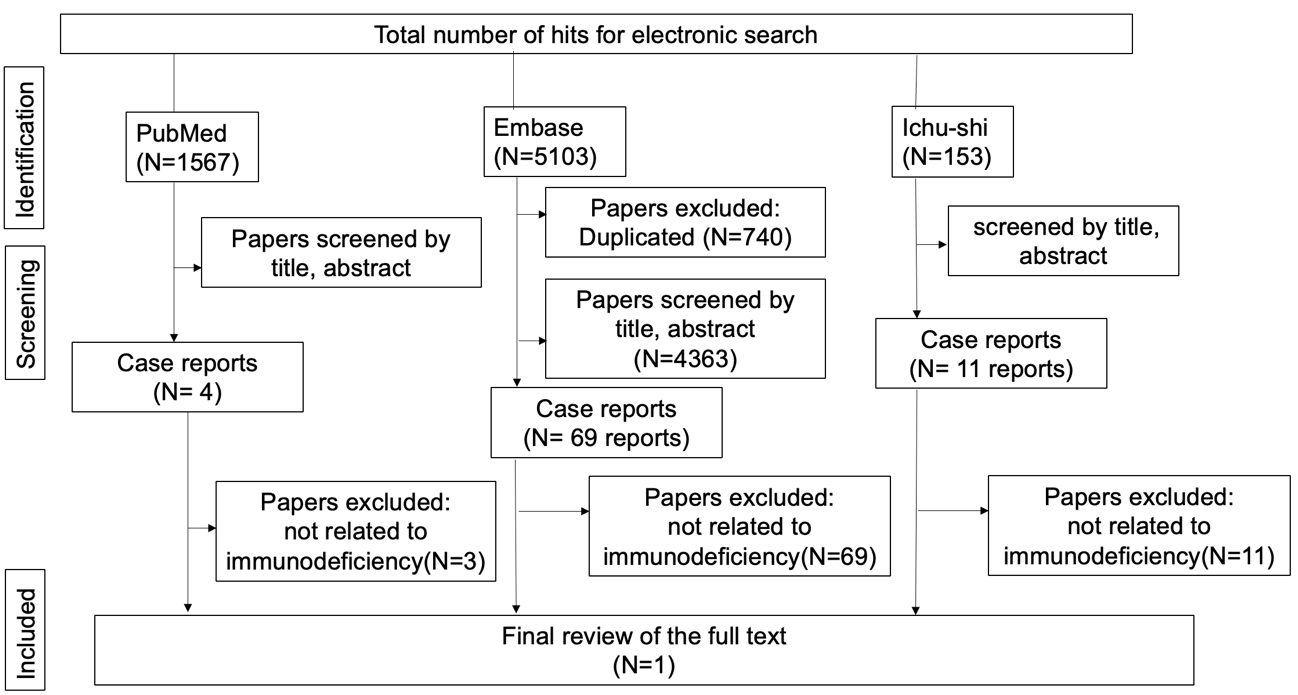


Table 1. Characteristics of Immunodeficiency Complications While Taking CDK4/6 Inhibitors

|  | Current case (68 y.o. F) | Case 1 (72 y.o. F) (Guillaume et al. 2020) | Case 2 (65 y.o. F) (Ashraf et al. 2022) |  |
| --- | --- | --- | --- | --- |
| Past chemotherapy | Cyclophosphamide, Epirubicin, Fluorouracil (17 years ago) | Taxan, bevacizumab  (8 years ago) | Doxorubicin, cyclophosphamide, and paclitaxel (5 years ago) |  |
| CDK4/6 inhibitor | Palbociclib (from 4 years ago) | Palbociclib (from 6 months ago) | Abemaciclib (from 1 month ago) |  |
| Immunosuppression related events | PML | Respiratory failure due to PCP (pneumocystis PCR positive with bronchoscopy specimen) | Respiratory failure due to unknown causal bacteria, treated as PCP |  |
| CD4+ T cell counts | 450/μL | <200/mm^3^ | No information |  |
| Lymphocyte counts | 600-800/μL | <1g/L | 1.9 k/mm^3^ |  |
| Serum IgG level | 829 mg/dL | No information | 163 mg/dL |  |
| * PCR; polymerase chain reaction | | | | |

A literature search was conducted in both the PubMed and Embase and ichushi databases (up to July 2024) using the following search terms:

For pubmed

| #4 | #3 and ("2000/01/01"[Date - Create] : "3000"[Date - Create]) AND ("english"[Language] OR "japanese"[Language]) |
| --- | --- |
| #3 | #1 and #2 |
| #2 | Opportunistic Infections[mh] OR Opportunistic Infection*[tiab] OR "Immunologic Deficiency Syndromes"[mh] OR immunodeficiency[tiab] OR Immunological Deficienc*[tiab] OR "cryptococcosis"[mh] OR Cryptococcosis infection*[tiab] OR Buschke disease[tiab] OR Busse-Buschke disease[tiab] OR "C. gattii Infection"[tiab] OR "C. neoformans infection"[tiab] OR "European blastomycosis"[tiab] OR Torulosis[tiab] OR Toruloses[tiab] OR "histoplasmosis"[mh] OR histoplasm*[tiab] OR cave disease[tiab] OR darling disease[tiab] OR "Mycobacterium Infections, Nontuberculous"[mh] OR "Mycobacterium Infection"[tiab:~3] OR "Atypical Mycobacteriosis Familial"[tiab:~3] OR "Atypical Mycobacterial Infection"[tiab:~3] OR ntm infection[tiab] OR "Papillomavirus Infections"[mh] OR HPV[tiab] OR Human Papilloma Virus*[tiab] OR human papillomavirus*[tiab] OR "Papillomaviridae"[mh] OR "Uterine Cervical Neoplasms"[mh] OR cervical cancer[tiab] OR cervical carcinoma[tiab] OR cervical neoplas*[tiab] OR cervical tumor[tiab] OR cervix cancer[tiab] OR cervix carcinoma[tiab] OR cervix neoplas*[tiab] OR cervix tumor[tiab] OR "Varicella Zoster Virus Infection"[mh] OR varicella-zoster virus[tiab] OR Congenital Varicella Syndrome[tiab] OR "Herpes simplex"[mh] OR Herpes Simplex infection*[tiab] OR herpes virus[tiab] OR "Oncogenic Viruses"[mh] OR oncogenic virus*[tiab] OR cancer virus*[tiab] OR tumor virus[tiab] OR "Sarcoma, Kaposi"[mh] OR Kaposi*[tiab] OR Multiple Idiopathic Pigmented Hemangiosarcoma[tiab] OR "Lymphoma, Non-Hodgkin"[mh] OR "non Hodgkin lymphoma"[tiab:~3] OR "Nonhodgkins Lymphoma"[tiab:~3] OR "Lymphoma, Diffuse"[tiab:~3] OR "Sarcoma, Lymphatic"[tiab:~3] OR Lymphosarcoma*[tiab] OR "Lymphoma Low Grade"[tiab:~3] OR "Mixed Lymphoma"[tiab:~3] OR "Nasopharyngeal Carcinoma"[mh] OR nasopharyngeal carcinoma*[tiab] OR "Stomach Neoplasms"[mh] OR gastric carcinoma*[tiab] OR stomach cancer*[tiab] OR stomach neoplasms*[tiab] OR "Molluscum Contagiosum"[mh] OR molluscum contagiosum[tiab] OR "Cytomegalovirus Infections"[mh] OR "CMV end organ disease*"[tiab] OR Cytomegalovirus Infection*[tiab] OR CMV Infection[tiab] OR Cytomegalovirus Colitis[tiab] OR Cytomegalovirus Inclusion[tiab] OR CMV Inclusions[tiab] OR Salivary Gland Virus Disease[tiab] OR Cytomegalic Inclusion[tiab] OR Inclusion Disease*[tiab] OR "Pneumonia, Pneumocystis"[mh] OR Pneumocystis Jirovecii pneumonia[tiab] OR "coccidioidomycosis"[mh] OR Coccidioidomycosis[tiab] OR Coccidioides[tiab] OR Leukoencephalopathy, Progressive Multifocal[mh] OR Progressive Multifocal Encephal*[tiab] OR Progressive Multifocal Leukoencephal*[tiab] OR Progressive Multifocal Leuco Encephal*[tiab] OR JC Polyomavirus Encephal*[tiab] OR "JC leukoencephalopathy"[tiab:~3] OR JC virus Encephal*[tiab] OR PML[tiab] OR JCVE[tiab] |
| #1 | "Cyclin-Dependent Kinases"[mh] AND (inhibit*[tiab] OR "Protein Kinase Inhibitors"[mh]) OR "Cyclin-Dependent Kinases/antagonists and inhibitors"[Mesh] OR ("cyclin dependent kinase"[tiab:~3] OR "cyclin dependent kinases"[tiab:~3]) AND (inhibit*[tiab] OR "protein kinase inhibitors"[mh]) OR "CDK inhibitor"[tiab:~4] OR "CDK inhibitors"[tiab:~4] OR CDKI[tiab] OR CDKIs[tiab] OR palbociclib[tw] OR Ibrance[tiab] OR "PD 0332991"[tiab] OR "PD 332991"[tiab] OR PD0332991[tiab] OR PD332991[tiab] OR abemaciclib[tw] OR Verzenio[tiab] OR LY2835219[tiab] OR "LY 2835219"[tiab] OR LY2385219[tiab] OR ribociclib[tw] OR Kisqali[tiab] OR "LEE 011"[tiab] OR LEE011[tiab] OR trilaciclib[tw] OR cosela[tiab] OR "g1t 28"[tiab] OR g1t28[tiab] |

For Embase

| #21 | #19 NOT #20 |
| --- | --- |
| #20 | #19 AND ('conference abstract'/it OR 'conference paper'/it OR 'conference review'/it OR 'editorial'/it OR 'letter'/it OR 'preprint'/it) |
| #19 | #1 AND #17 AND ([english]/lim OR [japanese]/lim) AND [2000-2024]/py |
| #18 | #1 AND #17 |
| #17 | #2 OR #3 OR #4 OR #5 OR #6 OR #7 OR #8 OR #9 OR #10 OR #11 OR #12 OR #13 OR #14 OR #15 OR #16 |
| #16 | progressive multifocal leukoencephalopathy'/exp OR ((‘JC polyomavirus’ OR ‘JC virus’ OR ‘JCV’) NEAR/2 (‘encephalopathy’ OR ‘leu$oencephal*’)):ti,ab,kw OR (‘multifocal leu$oencephal*’):ti,ab,kw OR (‘progressive multifocal’ NEAR/2 ‘encephal*’):ti,ab,kw OR (‘progressive multifocal’ NEAR/2 ‘leu$oencephal*’):ti,ab,kw OR ‘PML’:ti,ab OR ‘JCVE’:ti,ab,kw |
| #15 | 'pneumocystis pneumonia'/exp OR 'interstitial plasma cell pneumonia':ti,ab,kw OR 'interstitial pneumocystic pneumonia':ti,ab,kw OR 'pcp pneumonia':ti,ab,kw OR 'pjp pneumonia':ti,ab,kw OR 'plasma cell pneumonia':ti,ab,kw OR 'pneumocystic carinii pneumonia':ti,ab,kw OR 'pneumocystic pneumonia':ti,ab,kw OR 'pneumocystis (carinii) jiroveci pneumonia':ti,ab,kw OR 'pneumocystis (pjp) pneumonia':ti,ab,kw OR 'pneumocystis carinii (pcp) pneumonia':ti,ab,kw OR 'pneumocystis carinii pneumonia':ti,ab,kw OR 'pneumocystis carinii pneumonitis':ti,ab,kw OR 'pneumocystis jiroveci pneumonia':ti,ab,kw OR 'pneumocystis jirovecii (pcp) pneumonia':ti,ab,kw OR 'pneumocystis jirovecii (pjp) pneumonia':ti,ab,kw OR 'pneumocystis jirovecii pneumonia':ti,ab,kw OR 'pneumoncystis carinii pneumonia':ti,ab,kw OR 'pneumonia, plasma cell':ti,ab,kw OR 'pneumonia, pneumocystis':ti,ab,kw OR 'pneumonia, pneumocystis carinii':ti,ab,kw OR 'pulmonary pneumocystosis':ti,ab,kw OR 'pneumocystis pneumonia':ti,ab,kw OR 'coccidioidomycosis'/exp OR 'c. immitis infection':ti,ab,kw OR 'c. posadasii infection':ti,ab,kw OR 'coccidioidal granuloma':ti,ab,kw OR 'coccidioides immitis infection':ti,ab,kw OR 'coccidioides infection':ti,ab,kw OR 'coccidioides posadasii infection':ti,ab,kw OR 'coccidioidomycoses':ti,ab,kw OR 'coccidioidomykosis':ti,ab,kw OR 'coccidioidosis':ti,ab,kw OR 'coccidioimycosis':ti,ab,kw OR 'coccidiomycoses':ti,ab,kw OR 'coccidiomycosis':ti,ab,kw OR 'desert rheumatism':ti,ab,kw OR 'granuloma, coccidioidal':ti,ab,kw OR 'infection by coccidioides immitis':ti,ab,kw OR 'ocular coccicioidomycosis':ti,ab,kw OR 'posada-wernicke disease':ti,ab,kw OR 'san joaquin valley fever':ti,ab,kw OR 'coccidioidomycosis':ti,ab,kw |
| #14 | 'molluscum contagiosum'/exp OR 'mollusca contagiosa':ti,ab,kw OR 'molluscum contagiosums':ti,ab,kw OR 'molluscum contagious':ti,ab,kw OR 'molluscum epitheliale':ti,ab,kw OR 'molluscum contagiosum':ti,ab,kw OR 'cytomegalovirus infection'/exp OR 'cmv disease':ti,ab,kw OR 'cmv infection':ti,ab,kw OR 'cmv infections':ti,ab,kw OR 'cmv syndrome':ti,ab,kw OR 'cmv-associated disease':ti,ab,kw OR 'congenital cytomegalic inclusion':ti,ab,kw OR 'congenital cytomegalic inclusion body disease':ti,ab,kw OR 'congenital cytomegalic inclusion disease':ti,ab,kw OR 'cytomegalic inclusion body disease':ti,ab,kw OR 'cytomegalic inclusion disease':ti,ab,kw OR 'cytomegalic infection':ti,ab,kw OR 'cytomegaloinfection':ti,ab,kw OR 'cytomegaloviral disease':ti,ab,kw OR 'cytomegaloviral infection':ti,ab,kw OR 'cytomegalovirus (cmv) infection':ti,ab,kw OR 'cytomegalovirus disease':ti,ab,kw OR 'cytomegalovirus infections':ti,ab,kw OR 'cytomegaly virus disease':ti,ab,kw OR 'cytomegaly virus infection':ti,ab,kw OR 'disease due to cytomegalovirus':ti,ab,kw OR 'infection by cmv':ti,ab,kw OR 'infection by cytomegalovirus':ti,ab,kw OR 'infection due to cytomegalovirus':ti,ab,kw OR 'salivary gland virus disease':ti,ab,kw OR 'cytomegalovirus infection':ti,ab,kw |
| #13 | 'nasopharynx carcinoma'/exp OR 'carcinoma of the epipharynx':ti,ab,kw OR 'carcinoma of the nasopharynx':ti,ab,kw OR 'carcinoma of the rhinopharynx':ti,ab,kw OR 'epipharyngeal carcinoma':ti,ab,kw OR 'epipharynx carcinoma':ti,ab,kw OR 'naso-pharyngeal carcinoma':ti,ab,kw OR 'nasopharyngeal carcinoma':ti,ab,kw OR 'postnasal space carcinoma':ti,ab,kw OR 'rhino-pharyngeal carcinoma':ti,ab,kw OR 'rhinopharyngeal carcinoma':ti,ab,kw OR 'rhinopharynx carcinoma':ti,ab,kw OR 'nasopharynx carcinoma':ti,ab,kw OR 'stomach tumor'/exp OR 'gastric mass (tumor)':ti,ab,kw OR 'gastric masses (tumor)':ti,ab,kw OR 'gastric neoplasia':ti,ab,kw OR 'gastric neoplasm':ti,ab,kw OR 'gastric subepithelial tumor':ti,ab,kw OR 'gastric tumor':ti,ab,kw OR 'gastric tumorigenesis':ti,ab,kw OR 'gastric tumour':ti,ab,kw OR 'mucosa tumor, stomach':ti,ab,kw OR 'mucosa tumour, stomach':ti,ab,kw OR 'neoplasia of the stomach':ti,ab,kw OR 'neoplasm of the stomach':ti,ab,kw OR 'neoplasms of the stomach':ti,ab,kw OR 'neoplastic gastric':ti,ab,kw OR 'neoplastic stomach':ti,ab,kw OR 'stomach mucosa tumor':ti,ab,kw OR 'stomach mucosa tumour':ti,ab,kw OR 'stomach neoplasia':ti,ab,kw OR 'stomach neoplasm':ti,ab,kw OR 'stomach neoplasms':ti,ab,kw OR 'stomach tumorigenesis':ti,ab,kw OR 'stomach tumour':ti,ab,kw OR 'stomach ulcerated tumor':ti,ab,kw OR 'stomach ulcerated tumour':ti,ab,kw OR 'stomach ulcerating tumor':ti,ab,kw OR 'stomach ulcerating tumour':ti,ab,kw OR 'tumor of the gastric':ti,ab,kw OR 'tumor of the stomach':ti,ab,kw OR 'tumor, stomach mucosa':ti,ab,kw OR 'tumour of the gastric':ti,ab,kw OR 'tumour of the stomach':ti,ab,kw OR 'tumour, stomach mucosa':ti,ab,kw OR 'stomach tumor':ti,ab,kw |
| #12 | 'non-hodgkin lymphoma'/exp OR 'lymphoma, non-hodgkin':ti,ab,kw OR 'non hodgkin lymphoma':ti,ab,kw OR 'non hodgkin malignant lymphoma':ti,ab,kw OR 'non hodgkin`s lymphoma':ti,ab,kw OR 'non hodgkin`s malignant lymphoma':ti,ab,kw OR 'non hodgkins lymphoma':ti,ab,kw OR 'non hodgkins malignant lymphoma':ti,ab,kw OR 'non-hodgkin malignant lymphoma':ti,ab,kw OR 'non-hodgkin`s lymphoma':ti,ab,kw OR 'non-hodgkin`s malignant lymphoma':ti,ab,kw OR 'non-hodgkins lymphoma':ti,ab,kw OR 'non-hodgkins malignant lymphoma':ti,ab,kw OR 'nonhodgkin lymphoma':ti,ab,kw OR 'nonhodgkin`s lymphoma':ti,ab,kw OR 'nonhodgkins lymphoma':ti,ab,kw OR 'non-hodgkin lymphoma':ti,ab,kw |
| #11 | 'kaposi sarcoma'/exp OR 'acrosarcoma, kaposi':ti,ab,kw OR 'angioendothelioma kaposi':ti,ab,kw OR 'angiogenic reticulosis':ti,ab,kw OR 'angiogenic reticulosis sarcoma':ti,ab,kw OR 'angiomatosis kaposi':ti,ab,kw OR 'angioreticuloendotheliosis':ti,ab,kw OR 'angioreticulomatosis cutanea':ti,ab,kw OR 'angiosarcomatosis kaposi':ti,ab,kw OR 'histioangioreticulosis kaposi':ti,ab,kw OR 'histioangioreticulosis, kaposi':ti,ab,kw OR 'idiopathic haemorrhagic sarcoma':ti,ab,kw OR 'idiopathic haemorrhagic sarcoma, kaposi':ti,ab,kw OR 'idiopathic hemorrhagic sarcoma':ti,ab,kw OR 'idiopathic hemorrhagic sarcoma, kaposi':ti,ab,kw OR 'kaposi acrosarcoma':ti,ab,kw OR 'kaposi angiosarcoma':ti,ab,kw OR 'kaposi disease':ti,ab,kw OR 'kaposi idiopathic haemorrhagic sarcoma':ti,ab,kw OR 'kaposi idiopathic hemorrhagic sarcoma':ti,ab,kw OR 'kaposi tumor':ti,ab,kw OR 'kaposi tumour':ti,ab,kw OR 'kaposi`s disease':ti,ab,kw OR 'kaposi`s sarcoma':ti,ab,kw OR 'kaposis sarcoma':ti,ab,kw OR 'karposi`s angiosarcoma':ti,ab,kw OR 'multiple haemorrhagic sarcoma':ti,ab,kw OR 'multiple hemorrhagic sarcoma':ti,ab,kw OR 'reticulosis angiogenic':ti,ab,kw OR 'rhadinovirus infection':ti,ab,kw OR 'sarcoma idiopathicum haemorrhagicum':ti,ab,kw OR 'sarcoma idiopathicum hemorrhagicum':ti,ab,kw OR 'sarcoma idiopathicum multiplex haemorrhagicum':ti,ab,kw OR 'sarcoma idiopathicum multiplex hemorrhagicum':ti,ab,kw OR 'sarcoma multiplex idiopathicum haemorrhagicum':ti,ab,kw OR 'sarcoma multiplex idiopathicum hemorrhagicum':ti,ab,kw OR 'sarcoma, kaposi':ti,ab,kw OR 'sarcoma, multiple haemorrhagic':ti,ab,kw OR 'sarcoma, multiple hemorrhagic':ti,ab,kw OR 'kaposi sarcoma':ti,ab,kw |
| #10 | 'tumor virus'/exp OR 'cancer virus':ti,ab,kw OR 'cancer viruses':ti,ab,kw OR 'cancerogenic virus':ti,ab,kw OR 'carcinogenic virus':ti,ab,kw OR 'carcinogenic viruses':ti,ab,kw OR 'oncogenetic virus':ti,ab,kw OR 'oncogenic virus':ti,ab,kw OR 'oncogenic viruses':ti,ab,kw OR 'oncogenous virus':ti,ab,kw OR 'oncovirus':ti,ab,kw OR 'tumor viruses':ti,ab,kw OR 'tumour virus':ti,ab,kw OR 'tumour viruses':ti,ab,kw OR 'tumor virus':ti,ab,kw |
| #9 | 'herpes simplex'/exp OR 'herpes':ti,ab,kw OR 'herpes simplex complex':ti,ab,kw OR 'herpes simplex disease':ti,ab,kw OR 'herpes simplex infection':ti,ab,kw OR 'herpes simplex viral infection':ti,ab,kw OR 'herpes simplex virus (hsv) infection':ti,ab,kw OR 'herpes simplex virus infection':ti,ab,kw OR 'herpes vulgaris':ti,ab,kw OR 'hsv infection':ti,ab,kw OR 'infection by herpes simplex':ti,ab,kw OR 'infection by herpes simplex virus':ti,ab,kw OR 'infection by hsv':ti,ab,kw OR 'infection caused by herpes simplex':ti,ab,kw OR 'infection caused by herpes simplex virus':ti,ab,kw OR 'infection caused by hsv':ti,ab,kw OR 'infection due to herpes simplex':ti,ab,kw OR 'infection due to herpes simplex virus':ti,ab,kw OR 'infection due to hsv':ti,ab,kw OR 'herpes simplex':ti,ab,kw |
| #8 | 'herpes zoster'/exp OR 'disseminated herpes zoster':ti,ab,kw OR 'herpes zona':ti,ab,kw OR 'herpes zoster disease':ti,ab,kw OR 'herpes zoster infection':ti,ab,kw OR 'herpes zoster neuralgia':ti,ab,kw OR 'herpes zoster paralysis':ti,ab,kw OR 'infection by varicella zoster virus':ti,ab,kw OR 'infection by vzv':ti,ab,kw OR 'infection caused by varicella zoster virus':ti,ab,kw OR 'infection caused by vzv':ti,ab,kw OR 'shingles':ti,ab,kw OR 'varicella zoster infection':ti,ab,kw OR 'varicella zoster viral infection':ti,ab,kw OR 'varicella zoster virus infection':ti,ab,kw OR 'varicellovirus infection':ti,ab,kw OR 'vzv infection':ti,ab,kw OR 'zoster':ti,ab,kw OR 'zoster, herpes':ti,ab,kw OR 'herpes zoster':ti,ab,kw OR 'congenital varicella syndrome':ti,ab,kw |
| #7 | 'uterine cervix tumor'/exp OR ((cervical NEAR/3 (cancer OR carcinoma OR neoplasm* OR tumo*)):ti,ab,kw) OR ((cervix NEAR/3 (cancer OR carcinoma OR neoplasm* OR tumo*)):ti,ab,kw) |
| #6 | 'papillomavirus infection'/exp OR 'hpv infection':ti,ab,kw OR 'hpv infections':ti,ab,kw OR 'hpv-16/18 infection':ti,ab,kw OR 'human papilloma virus infection':ti,ab,kw OR 'human papillomavirus 16/18 infection':ti,ab,kw OR 'human papillomavirus infection':ti,ab,kw OR 'infection by hpv':ti,ab,kw OR 'infection by human papilloma virus':ti,ab,kw OR 'infection by human papillomavirus':ti,ab,kw OR 'infection caused by hpv':ti,ab,kw OR 'infection caused by human papillomavirus':ti,ab,kw OR 'infection with hpv':ti,ab,kw OR 'infection with human papillomavirus':ti,ab,kw OR 'papilloma viral infection':ti,ab,kw OR 'papillomaviral infection':ti,ab,kw OR 'papillomaviral infections':ti,ab,kw OR 'papillomavirus infections':ti,ab,kw OR 'papillomavirus infection':ti,ab,kw |
| #5 | 'atypical mycobacteriosis'/exp OR 'atypical mycobacteria infection':ti,ab,kw OR 'atypical mycobacterial disease':ti,ab,kw OR 'atypical mycobacterial infection':ti,ab,kw OR 'atypical mycobacterium infection':ti,ab,kw OR 'atypical mycobacterium infections':ti,ab,kw OR 'mycobacteriosis, atypical':ti,ab,kw OR 'mycobacterium infections, atypical':ti,ab,kw OR 'mycobacterium infections, nontuberculous':ti,ab,kw OR 'non tuberculous mycobacteria infection':ti,ab,kw OR 'non tuberculous mycobacterial infection':ti,ab,kw OR 'non tuberculous mycobacterium infection':ti,ab,kw OR 'non-tuberculous mycobacteria infection':ti,ab,kw OR 'non-tuberculous mycobacterial infection':ti,ab,kw OR 'non-tuberculous mycobacterium infection':ti,ab,kw OR 'nontuberculous mycobacteria infection':ti,ab,kw OR 'nontuberculous mycobacterial infection':ti,ab,kw OR 'nontuberculous mycobacterium infection':ti,ab,kw OR 'nontuberculous mycobacterium infections':ti,ab,kw OR 'tuberculoid cutaneous infection':ti,ab,kw OR 'atypical mycobacteriosis':ti,ab,kw OR 'ntm infection*':ti,ab,kw |
| #4 | 'histoplasmosis'/exp OR 'cave disease':ti,ab,kw OR 'darling disease':ti,ab,kw OR 'darling`s disease':ti,ab,kw OR 'h. capsulatum infection':ti,ab,kw OR 'histo-plasmosis':ti,ab,kw OR 'histoplasma capsulatum infection':ti,ab,kw OR 'histoplasma infection*':ti,ab,kw OR 'histoplasmoses':ti,ab,kw OR 'infection by h. capsulatum':ti,ab,kw OR 'infection by histoplasma':ti,ab,kw OR 'infection by histoplasma capsulatum':ti,ab,kw OR 'infection caused by histoplasma capsulatum':ti,ab,kw OR 'infection due to histoplasma capsulatum':ti,ab,kw OR 'histoplasmosis':ti,ab,kw |
| #3 | 'cryptococcosis'/exp OR ((cryptococcus NEAR/2 infection*):ti,ab,kw) OR 'buschke disease':ti,ab,kw OR 'busse buschke disease':ti,ab,kw OR 'c. gattii infection':ti,ab,kw OR 'c. neoformans infection*':ti,ab,kw OR 'european blastomycosis':ti,ab,kw OR 'torulos$s':ti,ab,kw |
| #2 | 'opportunistic infection'/exp OR 'opportunistic infection*':ti,ab,kw OR 'immune deficiency'/exp OR ((immun* NEAR/2 (defic* OR depress* OR incompetenc*)):ti,ab,kw) OR immunodeficiency:ti,ab,kw OR immunodepression:ti,ab,kw OR immunosuppression:ti,ab,kw |
| #1 | 'cyclin dependent kinase inhibitor'/exp OR ((cycli* NEAR/3 dependent NEAR/3 inhibitor*):ti,ab,kw) OR (('cdk' NEAR/3 inhibitor*):ti,ab,kw) OR cdki:ti,ab,kw OR cdkis:ti,ab,kw OR 'palbociclib'/exp OR palbociclib:ti,ab,kw,tn OR ibrance:ti,ab,kw,tn OR 'pd 0332991':ti,ab,kw,tn OR 'pd 332991':ti,ab,kw,tn OR 'pd0332991':ti,ab,kw,tn OR 'pd332991':ti,ab,kw,tn OR 'abemaciclib'/exp OR verzenio:ti,ab,kw,tn OR ly2835219:ti,ab,kw,tn OR 'ly 2835219':ti,ab,kw,tn OR ly2385219:ti,ab,kw,tn OR 'ribociclib'/exp OR kisqali:ti,ab,kw,tn OR 'lee 011':ti,ab,kw,tn OR lee011:ti,ab,kw,tn OR 'trilaciclib'/exp OR cosela:ti,ab,kw,tn OR 'g1t 28':ti,ab,kw,tn OR g1t28:ti,ab,kw,tn |

For ichushi

| #4 | (#3) and (DT=2000:2024) |
| --- | --- |
| #3 | #1 and #2 |
| #2 | opportunistic infection[Japanese]/TH or opportunistic infection[Japanese]/TA or immunodeficiency syndrome[Japanese]/TA or T-cell congenital anomaly[Japanese]/TA or antibody deficit syndrome[Japanese]/TA or antibody deficiency symptom[Japanese]/TA or antibody deficiency syndrome[Japanese]/TA or immunodeficiency syndrome[Japanese]/TA or cryptococcosis[Japanese]/TH or kuriputokokkasu[Japanese]/TA or kuriputokokkusu[Japanese]/TA or Histoplasmosis[Japanese]/TH or Dahling's disease[Japanese]/TA or Histoplasma[Japanese]/TA or hikekkakuseikousannkinnsyou[Japanese]/TH or hikekkakukinnseikousannkinkansensyou[Japanese]/TA or hikekkakukinseikousannkinnsyou[Japanese]/TA or hiteikei mikobakuteriaru infection(kana)[Japanese]/TA or hiteikei kousannseikinnsyou[Japanese]/TA or Tuberculosis-like disease[Japanese]/TA or Papillomaviridae[Japanese]/TH or Papillomavirus[Japanese]/TA or Cervical Tumor[Japanese]/TH or uterine cervix cancer[Japanese]/TA or cervical cancer[Japanese]/TA or Varicella Zoster Virus Infection[Japanese]/TH or Varicella[Japanese]/TA or Congenital Varicella Syndrome[Japanese]/TA or Herpes Simplex[Japanese]/TH or Herpes Simplex infection[Japanese]/TA or HSV Infection[Japanese]/TA or Neonatal Herpes[Japanese] /TA or Herpes Simplex[Japanese]/TA or tanjunsei herupesu[Japanese]/TA or tanjunsei housin[Japanese]/TA or Herpes Simplex Virus Infection[Japanese]/TA or Febrile Vesicular Eruption[Japanese]/TA or Tumor Virus[Japanese]/TH or Cancer Virus[Japanese]/TA or Oncogenic Virus[Japanese]/TA or Tumorigenic Virus[Japanese]/TA or Carcinogenic virus[Japanese]/TA or oncovirus[Japanese]/TA or gan(hirakana)hassei Virus[Japanese]/TA or gan(kana)hassei virus [Japanese]/TA or tumorgenesis virus[Japanese]/TA or syuyougensei viru(kanji)/TA or hatsugan(hirakana) virus[Japanese]/TA or hatsugan(kana) virus[Japanese]/TA or hatugansei(kanji) virus[Japanese] or Kaposi(english) sarcoma[Japanese]/TH or Kaposi sarcoma[Japanese]/TA or Kaposi's sarcoma[Japanese]/TA or Hemorrhagic sarcoma[Japanese]/TA or Idiopathic multiple pigmented sarcoma[Japanese]/TA or Non-Hodgkin lymphoma[Japanese]/TH or Lymphoma[Japanese]/TA or Oropharynx gan(kanji)[Japanese]/TH or Oropharynal gan(hirakana) [Japanese]/TA or Oropharynx gan(kana) [Japanese]/TA or Nasopharyngeal gan(kanji)[Japanese]/TA or Nasopharyngeal gan(hirakana) [Japanese]/TA or Nasopharyngeal gan(kana) [Japanese]/TA or Nasopharynx gan(hirakana)[Japanese]/TA or Nasopharynx gan(kana) [Japanese]/TA or Nasopharynx gan(kanji) [Japanese]/TA or Nasopharynx part of gan(hirakana) [Japanese]/TA or Nasopharynx part of gan(kana) [Japanese]/TA or Nasopharynx part of gan(kanji) [Japanese] or Stomach tumor[Japanese]/TH or gastric gan(kanji)[Japanese]/TA or gastric gan(hirakana)[Japanese] /TA or Tumor in stomach[Japanese] /TA or Stomach gam(kana)[Japanese]/TA or Stomach primary cancer[Japanese] /TA or Stomac malignant tomor[Japanese]/TA or Early-stage gastric cancer(kana)[Japanese] /TA or Early-stage gastric cancer(hirakana)[Japanese] /TA or Early-stage gastric cancer(kanji)[Japanese] /TA or Gastric submucosal tumor[Japanese]/TA or Molluscum contagiosum[Japanese]/TH or mizuibo(hirakana)[Japanese]/TA or Molluscum contagiosum [Japanese]/TA or water ibo(hirakana) [Japanese]/TA water ibo(kana) [Japanese]/TA or dennsennsei nann ibo(hirakana)[Japanese]/TA or dennsennsei nanyou(hirakana)[Japanese]/TA or dennsennsei nannibo(kanji)[Japanese]/TA or nanibo(hirakana)[Japanese]/TA or nannibo(kanji)[Japanese]/TA or Subcutaneous condyloma[Japanese]/TA or Cytomegalovirus Infectious disease[Japanese]/TH or Cytomegalovirus infection[Japanese]/TA or kyosaibouseifuunyuutaibyou[Japanese] /TA or kyosaibouseifuunyuutaisyou[Japanese] /TA or kyosaiboufuunyuutaibyou[Japanese]/TA or neuronal intranuclear inclusion disease[Japanese]/TA or Salivary Gland Virus Disease[Japanese]/TA or Pneumonia - Pneumocystis[Japanese]/TH or Pneumocystis pneumonia[Japanese]/TA or kokushijioides shou [Japanese]/TH or kokushijioido shou[Japanese]/TA or Valley Fever[Japanese]/TA or Leukoencephalopathy - Progressive Multifocal[Japanese]/TH or Progressive Multifocal Leukoencephalopathy[Japanese]/TA |
| #1 | Cyclin-Dependent/AL and Phosphotransferases/TH or (CDK/TA and inhibition[Japanese]/TA) or Cyclin-Dependent Kinases[Japanese]/TA or “cdk Protein(kana)[Japanese]”/TA or “cdk protein(kanji)[Japanese]”/TA or Palbociclib/TH or palbociclib/TA or palbocyclib[Japanese]/TA or palbociclib[Japanese]/TA or abemaciclib/TH or abemaciclib/TA or abemaciclib[Japanese]/TA or **Verzenio**[Japanese]/TA or Ribociclib/TH or Ribociclib/TA or ribociclib[Japanese]/TA or Trilaciclib/TH or Trilaciclib/TA or trilaciclib[Japanese]/TA |

Supplement. Lisco A, Ortega-Villa AM, Mystakelis H, et al (2023) Reappraisal of Idiopathic CD4 Lymphocytopenia at 30 Years. N Engl J Med 388:1680–1691
